# Multivariate analysis identifying the main factors associated with cow productivity and welfare in tropical smallholder dairy farms in Vietnam

**DOI:** 10.1007/s11250-022-03303-7

**Published:** 2022-09-22

**Authors:** Nguyen N. Bang, Nguyen V. Chanh, Nguyen X. Trach, Duong N. Khang, Ben J. Hayes, John B. Gaughan, Russell E. Lyons, David M. McNeill

**Affiliations:** 1 School of Veterinary Science, The University of Queensland, Gatton, Qld 4343 Australia; 2 Faculty of Animal Science, Vietnam National University of Agriculture, Hanoi, 131000 Vietnam; 3 Faculty of Animal Science and Veterinary Medicine, Nong Lam University, Ho Chi Minh, 71308 Vietnam; 4 Queensland Alliance for Agriculture and Food Innovation, The University of Queensland, St Lucia, Qld 4067 Australia; 5 School of Agriculture and Food Sciences, The University of Queensland, Gatton, Qld 4343 Australia; 6 School of Environmental and Rural Science, University of New England, Armidale, NSW 2351 Australia

**Keywords:** Bayesian modelling, Productivity, Welfare, Dairy cow, Small holder

## Abstract

This study aimed to rank potential drivers of cow productivity and welfare in tropical smallholder dairy farms (SDFs) in Vietnam. Forty-one variables were collected from 32 SDFs located in four geographically diverse dairy regions, with eight SDFs per region. Twelve variables, including milk yield (MILK), percentages of milk fat (mFA), protein (mPR), dry matter (mDM), energy-corrected milk yield (ECM), heart girth (HG), body weight (BW), ECM per 100 kg BW (ECMbw), body condition score (BCS), panting score (PS), inseminations per conception (tAI), and milk electrical resistance (mRE) of cows, were fitted as outcome variables in the models. Twenty-one other variables describing farm altitude, housing condition, and diet for the cows, cow genotypes, and cow physiological stage were fitted as explanatory variables. Increased farm altitude was associated with increases in ECM and mRE and with decreases in PS and tAI (*P* < 0.05). Increases in roof heights and percentage of shed side open were associated with increases in ECM, mFA, and mDM (*P* < 0.05). Increased dry matter intake and dietary densities of dry matter and fat were associated with increased MILK, ECM, and ECMbw and decreased tAI (*P* < 0.05). Increased dietary lignin density was associated with increased PS. Increased genetic proportion of Brown Swiss in the herd was associated with increased MILK, ECM, and ECMbw (*P* < 0.05). Thus, to improve cow productivity and welfare in Vietnamese SDFs, the following interventions were identified for testing in future cause-effect experiments: increasing floor area per cow, roof heights, shed sides open, dry matter intake, dietary fat density, and the genetic proportion of Brown Swiss and decreasing dietary lignin density.

## Introduction

Smallholder dairy farms (SDFs) are the most popular type of dairy farming system in tropical developing countries in South East Asia (SEA), including Vietnam (Devendra, 2001; Moran, 2015). For example, in Vietnam, approximately 28,695 SDFs were raising an average of 20 cows per farm or less and accounting for 80% of the national fresh milk production in 2016 (General Statistics Office of Vietnam, 2016; Nguyen et al., 2016; Vinamilk, 2017). Currently, there are relatively limited studies on SDFs in SEA, and the majority of the available studies on SDFs are limited to describing basic characteristics such as cow numbers, herd structure, and farmer-reported milk yields (Moran and Brouwer, 2013). Common characteristics of SDFs across SEA countries are low milk production relative to both genetic potential and cows under similar environmental conditions in developed countries: poor welfare as shown by panting in response to heat stress, low fertility, and high incidence of mastitis (Lam et al., 2010; Moran and Brouwer, 2013; Östensson et al., 2013; Moran and Morey, 2015). The few studies that have estimated average individual milk yields indicate that it is 14 to 15 kg/cow/d for cows in some northern provinces (Ashbaugh, 2010; Vu et al., 2016) of Vietnam, 8.3 to 15.3 kg/cow/d in Indonesia (Moran and Doyle, 2015), and 12.4 to 14.1 kg/cow/d in Thailand (Koonawootrittriron et al., 2009; Wongpom et al., 2017).

Finding the reasons for the low productivity and welfare of SDF cows is important but complicated because SDFs, like any agricultural system, are multidimensional and complex systems where many factors, including weather, shed conditions, nutrition, and genotype, simultaneously influence cow productivity and welfare. It is reasonable to propose that the low productivity and welfare of the cows in SDFs in SEA prevail for the following reasons. Firstly, the hot and humid weather conditions in SEA are not conducive to raising high-producing dairy cows. Secondly, the dairy cattle in SEA are commonly crossbreeds between internationally popular dairy breeds, mainly Holsteins (HOL), with local multipurpose Zebu breeds (ZEB). These crossbreeds might adapt well to the local conditions but be less productive than the pure dairy breeds such as HOL, Brown Swiss (BSW), or Jersey (JER) (Koonawootrittriron et al., 2009; Lam et al., 2010; Wongpom et al., 2017). Thirdly, the low quality of tropical roughage and by-product types commonly used by farmers, such as Napier grass (Pennisetum purpureum) or rice straw, and simple diet formulation such as roughage plus concentrate pellets added at a ratio of 1 kg per 2 kg of expected milk yield, does not supply enough or an appropriate balance of nutrients for the cows (Chu et al., 2005; Cuong et al., 2006; Lam et al., 2010; Moran, 2012; Pilachai et al., 2013; Bernard, 2015; Hiep et al., 2016; Phong and Thu, 2016). Furthermore, the poor design of the cowsheds, including a lack of cow-cooling facilities, most likely makes the cows uncomfortable and less productive (Chu et al., 2005; Moran and Brouwer, 2013; Phong and Thu, 2016).

While all the above-mentioned reasons can simultaneously serve to lower the productivity and welfare of cows, some might be more important than others. Identification of those that have the greatest impact on herd productivity and welfare is of practical importance as it can help to define the most needed short-term and long-term interventions. Mathematically, if all the input management and output data of the SDFs are available, the key drivers of cow productivity and welfare can be identified by building multivariate models which include only factors with significant effects. However, this type of study on SDFs is currently very limited because of the lack of accurate and complete dairy farming data. SDF farmers in SEA commonly do not record the production data properly. For example, a study in Thailand reported that 70% of all farms in the survey did not record production data (Yeamkong et al., 2010). Currently, the common strategies that farmers are applying to improve cow productivity appear to focus on genetic and nutritional aspects of herd management. Backcrossing current dairy herds with imported dairy sires of high genetic merit, mainly HOL, and directly importing HOL heifers, are common genetic strategies (Lam et al., 2010; Moran, 2015). Buying high-quality commercial concentrated pellets rich in protein and energy, and offering them at least twice a day to complement the balance of nutrients in basal forage, is the common nutritional strategy (Lam et al., 2010). Choosing highland rather than lowland regions to develop dairy farms was also a common strategy in the past, but currently, this is limited as the demand for milk in the cities, all in hot lowland areas, is rapidly increasing, while the availability of affordable land in the highlands is rapidly decreasing. Dairying in the lowlands is also considered to be more attractive than in the highlands as fresh milk can be delivered to market more cheaply and more reliably in terms of quality assurance (Su and Binh, 2001; Cai and Long, 2002; Moran and Morey, 2015; Herawati et al., 2016).

In this study, we focused on the multidimensionality of Vietnamese SDFs. The aim was to combine the data from the different themes of previous studies by the same authors (Bang et al., 2021b, 2021a, 2021c, 2021d) to build multivariate explanatory models to enable the identification and ranking of potential factors affecting the productivity and welfare of dairy cows in SDFs. The factors include milk yield and quality, body weight and body condition score, heat stress level, fertility, and udder health of the cows. We hypothesized that current improvement strategies found to varying extents in 32 SDF herds, such as increased altitude, infusion of HOL genetics, and increased dietary energy and protein concentrations, are all associated with the improvement of some key productivity and welfare indicators of the cows.

## Materials and methods

### Study sites, time, and data 

The study was conducted from 24 August to 7 October 2017 on 32 SDFs selected randomly from four key dairy regions of Vietnam, including a southern lowland, a southern highland, a northern lowland, and a northern highland region, with eight farms per region. To ensure the representativity of the data, firstly, the lists of SDFs in each region were obtained by contacting the regional District Agriculture Departments. Then, 40 SDFs per region, which account for 5 to 20% of total SDFs in each region, were randomly chosen from those lists to be included in a collaborative economic survey of SDFs (Nga, 2017a; b). Finally, eight SDFs per region were randomly selected from the SDFs in that economic survey who agreed to continued involvement in further studies. More details about the process to select the SDFs for the current study can be found in our previous studies (Bang et al., 2021c, 2021b, 2021a, 2021d).

Each farm was visited over a 24-h period to collect the necessary data. Microclimate data from 0600 to 1800 h within the cowshed of each SDF was measured by a Kestrel 5400 Heat Stress Tracker (NIELSEN-KELLERMAN, USA). Means (± SD) of ambient temperature, humidity, and temperature-humidity index (THI) in South Lowland were 29.5 ± 1.1°C, 81.8 ± 3.8%, and 82.5 ± 1.3 units, respectively; in South Highland, 25.4 ± 1.3°C, 80.5 ± 4.9%, and 75.5 ± 1.6 units; in North Lowland, 29.7 ± 1.5°C, 82.0 ± 6.0%, and 82.9 ± 2.0 units; and in North Highland, 26.0 ± 1.1°C; 80.6 ± 4.1%, and 76.7 ± 1.3 units (Bang et al., 2021c).

A total of 41 variables were used (Table 1). These variables were obtained from previous studies which were conducted concurrently on the same SDFs and by the same authors (Bang et al., 2021c, 2021b, 2021a, 2021d). In Table 1, to assist with the interpretation of the models and results, these variables were divided into three groups. Firstly, housing management; these were variables 1 to 7, from a housing management study (Bang et al., 2021c). Secondly, nutrition; these were variables 8 to 23, from a nutrition study (Bang et al., 2021a). Thirdly, animal; these were variables 24 to 41, from a cow welfare and productivity study (Bang et al., 2021b) and a cow genetic study (Bang et al., 2021d). The animal variables were grouped into three subgroups, including genetic (variables 24 to 27), physiological stage (variables 28 and 29), and productivity and welfare (variables 30 to 41). All cow-level variables were average per farm to obtain farm-level variables. Then, only farm-level variables were used to build the models.

**Table 1:** Summary of the studied variables^A^

No	Variable, unit	Explanation	Mean	SD	Min	Max	Variable
1	Altitude, m	Altitude	495	464	5	990	Predictor
2	FloorCow, m^2^/cow	Floor area per cow	8.7	4.3	3.1	21.3	Predictor
3	MatCow, m^2^/cow	Mat area per cow	1.2	1.1	0.0	4.0	Predictor
4	RidgeHei, m	Ridge roof height	3.7	0.8	2.7	6.0	Predictor
5	EaveHei, m	Eave roof height	2.8	0.6	2.1	4.2	Predictor
6	SideOpen, %	Per cent of shed sides open	75	21	25	100	Predictor
7	FanCow, fans/cow	Number of fans per cow	0.2	0.3	0.0	1.0	Predictor
8	DMIbw, % BW	Dry matter intake per body weight	3.2	0.3	2.6	3.9	Predictor
9	DM, %	Dietary dry matter concentration	35.9	4.0	27.5	44	Predictor
10	NEL, MCal/kg DM	Dietary net energy for lactation	1.4	0.1	1.2	1.5	Predictor
11	CP, % DM	Dietary crude protein	16.5	1.7	12.7	21.1	Predictor
12	ADF, % DM	Dietary acid detergent fibre	27.3	2.7	22.1	35.2	Predictor
13	NDF, % DM	Dietary neutral detergent fibre	45.8	3.7	37.4	56.6	Predictor
14	Fat, % DM	Dietary fat	3.8	0.5	2.9	5.1	Predictor
15	Starch, % DM	Dietary starch	19.1	4.1	10.4	30.4	Predictor
16	NFC, % DM	Dietary non-fibre carbohydrate	27.4	4.0	16.6	37.6	Predictor
17	Lignin, % DM	Dietary lignin	6.0	0.9	4.7	8.7	Predictor
18	Ca, % DM	Dietary calcium	0.78	0.19	0.52	1.14	Predictor
19	K, % DM	Dietary potassium	1.31	0.27	0.85	1.94	Predictor
20	Mg, % DM	Dietary magnesium	0.29	0.06	0.22	0.42	Predictor
21	Na, % DM	Dietary sodium	0.30	0.08	0.21	0.51	Predictor
22	P, % DM	Dietary phosphorus	0.43	0.05	0.33	0.53	Predictor
23	S, % DM	Dietary sulphur	0.24	0.03	0.18	0.32	Predictor
24	ZEB, %	Average Zebu ancestry proportion	4.3	2.7	0.9	12.2	Predictor
25	HOL, %	Average Holstein ancestry proportion	84.7	7.4	54.9	95.0	Predictor
26	BRW, %	Average Brown Swiss ancestry proportion	4.9	1.6	1.7	7.9	Predictor
27	JER, %	Average Jersey ancestry proportion	6.1	6.2	0.0	32.2	Predictor
28	DIM, days	Days in milk	181	57	41	330	Predictor
29	Lactations	Number of lactations	2.2	0.5	1.0	4.0	Predictor
30	HG, cm	Heart girth	186	6	171	202	Outcome
31	BW, kg	Body weight	498	49	392	623	Either
32	BCS, 1 to 5	Body condition score	2.8	0.2	2.4	3.4	Either
33	MILK, kg/cow/d	Milk yield	16.8	3.6	9.2	23.7	Outcome
34	ECM, kg/cow/d	Energy-corrected milk	15.7	3.0	8.9	21.6	Outcome
35	ECMbw, kg/100 kg BW/d	ECM per 100kg of body weight	3.2	0.5	2.0	4.3	Outcome
36	mFA, %	Milk fat concentration	3.7	0.4	2.9	4.5	Outcome
37	mPR, %	Milk protein concentration	3.3	0.3	2.7	4.2	Outcome
38	mDM, %	Milk dry matter concentration	12.3	0.7	10.9	13.6	Outcome
39	mRE, units	Milk electrical resistance	406	25	362	477	Outcome
40	PS, 0 to 4.5	Panting score	1.3	0.5	0.5	2.4	Either
41	tAI, times	Inseminations per conception	2.3	1.2	1.0	6.0	Outcome

^A^Body condition score: 1 = thin to 5 = obese; Panting score: 0 = normal to 4.5 = extreme heat stressed

Variables 1 to 7 were from a housing management study (Bang et al., 2021c), 8 to 23 from a nutrition study (Bang et al., 2021a), 24 to 27 from a cow genetic study (Bang et al., 2021d), and 28 to 41 from a cow welfare and productivity study (Bang et al., 2021b)

### Building the models

#### Models

Milk production, panting score (PS), number of artificial inseminations (tAI), and milk electrical resistance (mRE) are productivity and welfare indicators because they reflect the status of production, heat stress, fertility, and udder health of the cows, respectively. High productivity, low PS, low tAI, and high mRE are preferable because they indicate that a cow is productive, not suffering from heat stress, fertile, and has good udder health. Since the aim was to build explanatory models of cow productivity and welfare indices, these indicators were chosen as outcome variables (or dependent variables), and the variables that theoretically reflect the causal structure of the outcome variables were chosen as the predictor (independent) variables (Shmueli, 2010) (Table 1). The management, nutrition, genetic, and physiological stage variables, all well-known as cow productivity and welfare drivers, were chosen as candidate predictor variables (NRC, 2001; Food and Agriculture Organization, 2011; USDA, 2016). The matrix notation describing the models was:

$$y=X\beta +e$$

where y was the vector of dependent variables, β was the vector of fixed effects (independent variables), e was the vector of residual random effects [assumed e ~ N(0, Iσ^2^_e_)], and X was the incidence matrices of the fixed effects.

#### Identification of initial independent variables

When building the models, firstly, multicollinearity was assessed using variance inflation factor (VIF) statistics. Predictor variables with VIF > 10 were excluded from the initial multivariate model (Kock and Lynn, 2012). Based on this procedure, 13 variables (Region, FanCow, NEL, CP, ADF, NDF, Starch, Ca, K, Mg, S, HOL, and HG) were excluded from all models. Table 2 summarizes the included variables.

**Table 2:** Summary of independent variables with variance inflation factor less than ten that were included in the initial models^A^

Outcome variables (y)	Predictor variables (or fixed effects β) with VIF < 10
MILK, ECM, ECMbw, mFA, mPR, mDM, mRE, or tAI	Altitude + FloorCow + MatCow + RidgeHei + EaveHei + SideOpen + DMIbw + DM + Fat + NFC + Na + ZEB + BRW + JER + DIM + Lactations + BW + BCS + PS
HG, BW, or BCS	Altitude + FloorCow + MatCow + RidgeHei + EaveHei + SideOpen + DMIbw + DM + Fat + NFC + Na + ZEB + BRW + JER + DIM + Lactations + Lignin + P + PS
PS	Altitude + FloorCow + MatCow + RidgeHei + EaveHei + SideOpen + DMIbw + DM + Fat + NFC + Na + ZEB + BRW + JER + DIM + Lactations + BW + BCS + Lignin + P

^A^Abbreviations: Altitude, (m); FloorCow, floor area per cow (m^2^/cow); MatCow, mat area per cow (m^2^/cow); RidgeHei, ridge roof height (m); EaveHei, eave roof height (m); SideOpen, per cent of shed sides open (%); DMIbw, dry matter intake per body weight (% BW); DM, dietary dry matter concentration (%); Fat, dietary fat (% DM); NFC, dietary non-fibre carbohydrate (% DM); Lignin, dietary lignin (% DM); Na, dietary sodium (% DM); P, dietary phosphorus (% DM); ZEB, average Zebu ancestry proportion (%); BRW, average Brown Swiss ancestry proportion (%); JER, average Jersey ancestry proportion (%); DIM, days in milk; Lactations, number of lactations; HG, heart girth (cm); BW, body weight (kg); BCS, body condition score (1 to 5); MILK, milk yield (kg/cow/d); ECM, energy-corrected milk (kg/cow/d); ECMbw, ECM per 100kg of body weight (kg/100 kg BW/d); mFA, milk fat concentration (%); mPR, milk protein concentration (%); mDM, milk dry matter concentration (%); mRE, milk electrical resistance (units); PS, panting score (0 to 4.5); tAI, inseminations per conception (times)

Since the excluded variables may still be correlated with explanatory and outcome variables included in the models, a Pearson correlation matrix was determined (Shmueli, 2010) (Table 3). Pearson correlations between variables included in the models are presented in Table 7 in the Appendix. In Table 3, correlations were only considered significant if they passed a Bonferroni corrected P-value. Farms with high altitude were associated with fewer fans per cow. Farms with high NFC were associated with low ADF, low NDF, and high Starch. Farms with high lignin concentration were associated with lower NEL and ADF. Farms with high P were associated with high CP and Ca. Farms with high JER were associated with low HOL. Magnesium was the only variable that was associated positively with MILK and ECM.

**Table 3:** Pearson correlations between variables included in the models (rows) and variables not included in the models due to variance inflation factor ≥ 10 (columns)^A^

Included variables^B^	Not included variables										
	FanCow	NEL	CP	ADF	NDF	Starch	Ca	K	Mg	S	HOL
Altitude	−0.66*	0.20	0.31	−0.06	−0.38	0.15	0.17	0.17	0.34	−0.27	0.43
FloorCow	−0.23	−0.41	0.07	0.43	0.10	−0.24	0.43	0.02	0.57	−0.03	0.31
MatCow	0.10	−0.31	−0.08	0.31	0.35	−0.2	0.02	−0.25	0.14	−0.05	0.18
RidgeHei	0.45	0.01	−0.27	−0.20	−0.24	0.27	0.28	−0.36	0.32	−0.09	0.03
EaveHei	0.59	−0.27	−0.38	0.08	0.04	0.06	0.33	−0.58	0.42	0.11	−0.14
SideOpen	−0.03	−0.17	−0.31	0.12	−0.15	0.25	0.05	0.07	0.23	−0.42	0.18
DMIbw	−0.02	0.10	−0.12	−0.15	−0.18	0.23	−0.01	−0.06	0.29	−0.15	−0.02
DM	0.06	0.32	0.12	−0.39	−0.47	0.09	0.80	−0.37	0.50	0.48	0.00
Fat	0.30	0.50	0.27	−0.47	−0.17	0.21	−0.25	−0.23	−0.31	0.09	−0.18
NFC	0.01	0.47	−0.36	−0.69*	−0.87***	0.90***	0.18	−0.16	0.08	−0.18	0.05
Lignin	−0.03	−0.65*	−0.06	0.68*	0.47	−0.52	0.29	−0.24	0.61	0.07	0.06
Na	−0.11	0.05	−0.14	0.08	−0.03	0.01	−0.01	−0.15	−0.03	−0.11	0.01
P	−0.41	0.32	0.77***	−0.24	−0.31	−0.3	0.69*	0.15	0.39	0.64	−0.08
ZEB	0.09	0.16	0.18	−0.10	0.20	−0.19	−0.39	0.19	−0.41	0.16	−0.41
BRW	−0.08	0.24	0.32	−0.19	−0.22	−0.03	0.23	−0.14	0.08	0.39	−0.48
JER	0.18	0.06	−0.08	−0.13	−0.03	0.07	0.04	−0.26	−0.15	0.48	−0.9***
DIM	0.19	−0.18	−0.07	0.08	0.09	−0.02	−0.03	0.06	−0.16	−0.17	0.20
Lactations	−0.30	0.14	0.12	−0.07	−0.28	0.1	0.14	0.16	0.31	−0.11	0.21
HG	0.12	−0.31	−0.14	0.3	0.07	−0.1	0.36	−0.09	0.4	−0.19	0.51
BW	0.12	−0.31	−0.15	0.29	0.05	−0.09	0.38	−0.11	0.42	−0.18	0.50
BCS	0.60	−0.08	−0.24	0.00	0.09	0.01	0.12	−0.22	0.04	0.04	0.06
MILK	−0.12	0.11	0.20	−0.10	−0.34	0.03	0.65	−0.26	0.70**	0.19	0.13
ECM	−0.05	0.11	0.18	−0.08	−0.32	0.00	0.64	−0.30	0.67*	0.22	0.05
ECMbw	0.01	0.25	0.21	−0.24	−0.37	0.07	0.54	−0.34	0.57	0.37	−0.26
mFA	0.35	−0.30	−0.15	0.32	0.48	−0.35	−0.26	0.10	−0.40	0.04	−0.19
mPR	0.50	−0.52	−0.31	0.43	0.49	−0.25	0.01	−0.26	−0.03	−0.05	0.02
mDM	0.26	−0.22	−0.22	0.25	0.44	−0.24	−0.43	0.04	−0.44	−0.02	−0.33
PS	0.15	−0.03	0.10	0.02	0.21	−0.32	0.15	−0.09	−0.05	0.57	−0.31
tAI	0.00	−0.12	−0.18	−0.02	0.09	0.08	−0.18	0.12	−0.43	0.05	−0.13
mRE	−0.23	0.11	−0.12	−0.14	−0.29	0.43	−0.13	0.03	0.08	−0.50	0.25

^A^Region was also not included in any models due to VIF > 10, but it is a categorical variable; thus, its correlations with other variables were unable to be tested. Significant levels were adjusted by the Bonferroni method: *P <0.05, **P <0.01 and ***P <0.001

^B^Abbreviations and units: Altitude (m); FloorCow, floor area per cow (m^2^/cow); MatCow, mat area per cow (m^2^/cow); RidgeHei, ridge roof height (m); EaveHei, eave roof height (m); SideOpen, per cent of shed sides open (%); DMIbw, dry matter intake per body weight (% BW); DM, dietary dry matter concentration (%); Fat, dietary fat (% DM); NFC, dietary non-fibre carbohydrate (% DM); Lignin, dietary lignin (% DM); Na, dietary sodium (% DM); P, dietary phosphorus (% DM); ZEB, average zebu ancestry proportion (%); BRW, average Brown Swiss ancestry proportion (%); JER, average Jersey ancestry proportion (%); DIM, days in milk; Lactations, number of lactations; HG, heart girth (cm); BW, body weight (kg); BCS, body condition score (1 to 5); MILK, milk yield (kg/cow/d); ECM, energy-corrected milk (kg/cow/d); ECMbw, ECM per 100kg of body weight (kg/100 kg BW/d); mFA, milk fat concentration (%); mPR, milk protein concentration (%); mDM, milk dry matter concentration (%); mRE, milk electrical resistance (units); PS, panting score (0 to 4.5); tAI, inseminations per conception (times); FanCow, number of fans per cow (fans/cow); NEL, dietary net energy for lactation (MCal/kg DM); CP, dietary crude protein (% DM); ADF, dietary acid detergent fibre (% DM); NDF, dietary neutral detergent fibre (% DM); Starch, dietary starch (% DM); Ca, dietary calcium (% DM); K, dietary potassium (% DM); Mg, dietary magnesium (% DM); S, dietary sulphur (% DM); HOL, average Holstein ancestry proportion (%)

#### Model building and model selection

After the initial independent variables were identified for each model, the Bayesian model averaging (BMA) method was applied to select the final multi-multivariate linear regression models using R package ‘BMA’ (Raftery et al., 2019). BMA method was chosen because it accounts for the uncertainty of variables included in the linear regression model by averaging over the best models identified, based on posterior model probability (Raftery et al., 2019). For each outcome variable, BMA method suggested the five best explanatory models, which were the ones with the highest coefficient of determination (R^2^) and the lowest Bayesian information criterion (BIC). Those best models included only the explanatory variables, which are suggestively (P ≤ 0.1) or significantly (P ≤ 0.05) associated with the outcome variables. Among the five best explanatory models for each outcome variable, only one explanatory model that either had highest R^2^ and lowest BIC or had the most biological meanings was selected to present. The final selected model was summarized by linear regression analysis to obtain regression coefficients ± SE and corresponding P-values. The final models were also further evaluated by looking at standardized residuals and leverage to ensure model assumptions were met (Richards et al., 2014). Specifically, the outliers, linearity of the data, normality of residuals, homoscedasticity, and independence of residuals error terms of models were checked by drawing diagnostic plots which visualize the residual errors of the models.

## Results

### Variables associated with milk production

The detailed models for each of the milk production outcome variables (Table 2) are presented in Table 4. The R^2^ was moderate for the mPR model (55%), high for mDM model (72%), and very high for other models (> 84% for the mFA, MILK, ECM and ECMbw models).

**Table 4:** Multivariate regression models identifying the variables affecting cow milk yield (MILK, kg/cow/d), milk fat (mFA, %), milk protein (mPR, %), milk dry matter (mDM, %), energy-corrected milk (ECM, kg/cow/d), and energy-corrected milk adjusted for body weight (ECMbw, kg/100kg BW/d)

	MILK		mFA		mPR		mDM		ECM		ECMbw	
Variables^A^	Coef (SE)^B^	*P*^C^	Coef (SE)	*P*	Coef (SE)	*P*	Coef (SE)	*P*	Coef (SE)	*P*	Coef (SE)	*P*
Intercept	−31.69 (5.09)	< 0.001	4.37 (0.74)	< 0.001	3.3 (0.55)	< 0.001	12.42 (0.92)	< 0.001	−35.96 (4.77)	< 0.001	−4.74 (0.79)	< 0.001
Altitude	--	--	--	--	−3E-4 (1E-4)	0.002	--	--	0.002 (0.001)	0.006	--	--
FloorCow	0.29 (0.06)	< 0.001	−0.03 (0.01)	0.002	--	--	−0.07 (0.02)	0.002	--	--	--	--
MatCow	−0.41 (0.2)	0.051	--	--	--	--	--	--	--	--	--	--
RidgeHei	--	--	0.18 (0.05)	0.002	--	--	--	--	0.67 (0.34)	0.060	--	--
EaveHei	--	--	--	--	--	--	0.31 (0.14)	0.031	--	--	--	--
SideOpen	--	--	0.009 (0.002)	< 0.001	--	--	0.013 (0.004)	0.009	0.040 (0.01)	0.007	0.008 (0.002)	0.004
DMIbw	5.08 (0.85)	< 0.001	−0.37 (0.16)	0.029	--	--	--	--	4.73 (0.82)	< 0.001	1.11 (0.15)	< 0.001
DM	0.30 (0.05)	< 0.001	−0.03 (0.01)	0.006	--	--	-0.10 (0.02)	< 0.001	0.26 (0.06)	< 0.001	0.05 (0.01)	< 0.001
Fat	1.58 (0.39)	< 0.001	--	--	--	--	--	--	1.05 (0.4)	0.016	0.32 (0.08)	< 0.001
NFC	--	--	−0.04 (0.01)	0.004	−0.03 (0.01)	0.014	--	--	−0.15 (0.06)	0.024	−0.03 (0.01)	0.029
Na	−6.53 (2.73)	0.026	1.68 (0.5)	0.003	--	--	2.76 (1.06)	0.016	--	--	--	--
ZEB	--	--	--	--	-0.07 (0.02)	< 0.001	--	--	--	--	-0.05 (0.02)	0.02
BRW	0.84 (0.13)	< 0.001	--	--	--	--	--	--	0.85 (0.13)	< 0.001	0.18 (0.03)	< 0.001
DIM	−0.011 (0.004)	0.007	0.002 (0.001)	0.018	--	--	--	--	--	--	--	--
Lactations	--	--	--	--	--	--	--	--	--	--	0.27 (0.08)	0.002
BW	0.04 (0.01)	< 0.001	--	--	--	--	--	--	0.03 (0.005)	< 0.001	--	--
BCS	−2.63 (1.09)	0.024	--	--	0.40 (0.17)	0.027	--	--	--	--	--	--
PS	--	--	0.61 (0.1)	< 0.001	--	--	1.11 (0.21)	< 0.001	1.34 (0.64)	0.049	0.28 (0.12)	0.024
R^2^, %	94		84		55		72		92		88	
Adj R^2^, %	91		78		49		66		88		83	
BIC	−58.3		−29.1		−11.9		−20.5		−45.0		−36.6	

^A^Abbreviations and units: Altitude (m); FloorCow, floor area per cow (m^2^/cow); MatCow, mat area per cow (m^2^/cow); RidgeHei, ridge roof height (m); EaveHei, eave roof height (m); SideOpen, per cent of shed sides open (%); DMIbw, dry matter intake per body weight (% BW); DM, dietary dry matter concentration (%); Fat, dietary fat (% DM); NFC, dietary non-fibre carbohydrate (% DM); Na, dietary sodium (% DM); P, dietary phosphorus (% DM); S, dietary sulphur (% DM); ZEB, average zebu ancestry proportion (%); BRW, average Brown Swiss ancestry proportion (%); DIM, days in milk; Lactations, number of lactations; BW, body weight (kg); BCS, body condition score (1 to 5); PS, panting score (0 to 4.5); MILK, milk yield (kg/cow/d); mFA, milk fat concentration (%); mPR, milk protein concentration (%); mDM, milk dry matter concentration (%); ECM, energy-corrected milk (kg/cow/d); ECMbw, ECM per 100kg of body weight (kg/100 kg BW/d); R^2^, coefficient of determination; Adj R^2^, adjusted coefficient of determination; BIC, Bayesian information criterion

^B^*Coef* coefficient; *SE* standard error

^C^The independent variables included in each model but had no significant effect (*P* > 0.10) included Average Jersey ancestry proportion and the variables with ‘--’ sign in P column

For housing management variables, increased per cent of shed sides open was associated with increases in the corrected milk yields (ECM and ECMbw) and milk components mFA and mDM but not mPR. Increased altitude was associated with increased ECM but decreased mPR. Increased floor area per cow was associated with increased MILK but decreased mFA and mPR. Increased ridge roof height was associated with increases in ECM and mFA. Increased eave height was associated with decreased mDM. Increased mat area per cow was associated with decreased MILK.

For nutritional variables, increases in dietary DMIbw, DM, and fat were associated with increases in MILK, ECM, and ECMbw. However, increased dietary DM was associated with decreases in mFA and mDM. Increased dietary Na was associated with decreased MILK (but not associated with ECM or ECMbw) and with increases in mFA and mDM. Increased dietary NFC was associated with decreases in mFA, ECM, and ECMbw.

For animal variables, higher BSW was associated with increases in MILK, ECM, and ECMbw. However, higher ZEB was associated with decreases in ECMbw and mPR. JER was not found to be associated with any milk yield or component variables. Increased BW was only associated with increased ECM. Increased DIM was associated with decreased MILK but with increased mFA. Increased number of lactations was associated with increased ECMbw. Increased BCS was associated with decreased MILK but with increased mPR.

Increased PS was associated with increases in mFA, mPR, ECM, and ECMbw.

### Variables associated with cow conformation

The models identifying the variables associated with body conformation showed moderate R^2^ ranging from 51 to 58% (Table 5). The variables associated with HG were also those associated with BW, which was to be expected as BW was estimated from HG. Increases in mat area per cow, eave height, and the number of lactations were associated with increases in both HG and BW. In contrast, increases in ZEB and JER were associated with decreases in both HG and BW. Increases in eave height, dietary fat, BRW, and PS were associated with increased BCS. In contrast, increases in dietary P and JER were associated with decreased BCS.

**Table 5:** Multivariate regression models identifying the variables affecting cow heart girth (HG, cm), body weight (BW, kg), and body condition score (BCS)

Variables^A^	HG		BW		BCS	
	Coef (SE)^B^	P^C^	Coef (SE)	P	Coef (SE)	P
Intercept	172.34 (5.82)	< 0.001	396.11 (43.71)	< 0.001	2.19 (0.4)	< 0.001
MatCow	1.68 (0.8)	0.046	12.66 (6.02)	0.045	--	--
EaveHei	3.11 (1.41)	0.036	23.77 (10.56)	0.033	0.14 (0.05)	0.012
Fat	--	--	--	--	0.10 (0.06)	0.089
P	--	--	--	--	−1.31 (0.7)	0.073
ZEB	−0.71 (0.31)	0.033	−5.64 (2.36)	0.025	--	--
BRW	--	--	--	--	0.04 (0.02)	0.057
JER	−0.37 (0.15)	0.019	−2.63 (1.12)	0.026	−0.02 (0.01)	0.005
Lactations	3.5 (1.67)	0.046	26.85 (12.53)	0.042	--	--
PS	--	--	--	--	0.22 (0.07)	0.004
R^2^, %	58		58		51	
Adj R^2^, %	50		50		40	
BIC	−10.3		−10.4		−2.5	

^A^Abbreviations and units: MatCow, mat area per cow (m^2^/cow); EaveHei, eave roof height (m); Fat, dietary fat (% DM); P, dietary phosphorus (% DM); ZEB, average zebu ancestry proportion (%); BRW, average Brown Swiss ancestry proportion (%); JER, average Jersey ancestry proportion (%); Lactations, number of lactations; PS, panting score (0 to 4.5); HG, heart girth (cm); BW, body weight (kg); BCS, body condition score (1 to 5); R^2^, coefficient of determination; Adj R^2^, adjusted coefficient of determination; BIC, Bayesian information criterion

^B^*Coef* coefficient; *SE* standard error

^C^The independent variables included in each model but had no significant effect included altitude, floor area per cow, ridge roof height, per cent of shed sides open, dry matter intake per body weight, dietary dry matter concentration (%), dietary non-fibre carbohydrate, dietary sodium, days in milk, lignin, and the variables with ‘--’ sign in P column

### Variables associated with the level of heat stress, reproduction, and udder health of the cows

The R^2^ was low for the mRE model (44%) but high for the PS model (75%) and tAI model (76%) (Table 6). A decreased tAI was associated with increases in altitude, DMIbw, dietary fat, and BW, and decreases in mat area per cow, NFC, and DIM. A decreased PS was associated with increases in altitude, eave height, and floor area per cow but with decreases in NFC, lignin, P, and BCS. An increase in mRE was associated with increases in altitude and DMIbw.

**Table 6:** Multivariate regression analysis identifying the variables affecting panting score (PS), times of artificial inseminations per conception (tAT, times), and milk electrical resistance (mRE, units)

Variables^A^	PS		tAI		mRE	
	Coef (SE)^B^	P^C^	Coef (SE)	P	Coef (SE)	P
Intercept	−3.35 (1.18)	0.009	12.89 (2.88)	< 0.001	304.48 (44.2)	< 0.001
Altitude	−0.0008 (0.0001)	< 0.001	−0.001 (0.0003)	0.034	0.03 (0.01)	< 0.001
FloorCow	−0.04 (0.02)	0.050	--	--	--	--
MatCow	--	--	0.49 (0.13)	< 0.001	--	--
EaveHei	−0.43 (0.10)	< 0.001	--	--	--	--
DMIbw	--	--	−1.27 (0.55)	0.030	26.6 (13.7)	0.062
Fat	--	--	−0.88 (0.23)	< 0.001	--	--
NFC	0.03 (0.02)	0.063	0.09 (0.03)	0.013	--	--
Lignin	0.35 (0.09)	< 0.001	--	--	--	--
P	4.53 (1.09)	< 0.001	--	--	--	--
DIM	--	--	0.008 (0.002)	0.003	--	--
BW	--	--	−0.015 (0.003)	< 0.001	--	--
BCS	0.6 (0.25)	0.023	--	--	--	--
R^2^, %	75		76		44	
Adj R^2^, %	68		69		40	
BIC	−20.5		−21.3		−11.3	

^A^Abbreviations and units: Altitude, (m); FloorCow, floor area per cow (m^2^/cow); MatCow, mat area per cow (m^2^/cow); EaveHei, eave roof height (m); DMIbw, dry matter intake per body weight (% BW); Fat, dietary fat (% DM); NFC, dietary non-fibre carbohydrate (% DM); Lignin, dietary lignin (% DM); P, dietary phosphorus (% DM); DIM, days in milk; BW, body weight (kg); BCS, body condition score (1 to 5); PS, panting score (0 to 4.5); tAI, inseminations per conception (times); R^2^, coefficient of determination; Adj R^2^, adjusted coefficient of determination; BIC, Bayesian information criterion

^B^*Coef* coefficient; *SE* standard error

^C^The independent variables included in each model but had no significant effect included ridge roof height, per cent of shed sides open, dietary dry matter concentration, dietary sodium, average zebu ancestry proportion, average Brown Swiss ancestry proportion, average Jersey ancestry proportion, lactations, dietary sulphur, and the variables with ‘--’ sign in P column. PS was not in the model for PS. Lignin and P were not in the models for tAI and mRE

## Discussion

This study aimed to build explanatory models to prioritize potential housing, nutrition, and animal variables affecting the productivity and welfare of dairy cows in SDFs. Except for the mRE model, with a relatively low R^2^ of 44%, the R^2^ of all other models ranged from moderate (51%) for BCS to very high (94%) for MILK, indicating that the important independent variables have been included in each model. Those important independent variables suggested a wide range of interventions worthy for the future research. The most inclusive models were for ECM and ECMbw, and since these indicators have already been corrected for variation in mFA and mPR (ECM) and BW (ECMbw), the variables affecting them deserve the most immediate attention. The variables most associated with ECM and ECMbw were altitude, ridge roof height, per cent of shed sides open, DMIbw, dietary DM concentration, dietary fat concentration, genetic proportions of BSW and ZEB, BW, and PS.

### Housing variables

Housing management variables, of all the variable groupings, were identified as the worthiest of future intervention research. Each 100-m increase in altitude was associated with an increase of 0.2 kg in daily ECM per cow, 0.08 unit decrease in mean PS, 0.1 times decrease in mean tAI, and 3 unit increase in mean mRE. These results are consistent with a case study of SDFs in Indonesia which reported that the daily milk yield of the lowland farms (8.3 kg/cow/d) was much lower than that of the highland farms (13.5 kg/cow/d), although this was only a simple comparison between regions without accounting for the effects of confounding variables associated with each region (Moran and Doyle, 2015).

Besides altitude, increased eave or ridge height of the cowshed roof was associated with increased ECM, mFA, mDM, HG, BW, and BCS and decreased PS, all of which are desirable. Similarly, increased floor area per cow was associated with increased MILK and decreased PS, and increased per cent of shed sides open was associated with an increased mFA and mDM and decreased PS. These associations are logical as altitude, roof height, floor area per cow, or per cent of shed sides open are all major variables affecting cow comfort by improving airflow, which should enhance the opportunity for evaporative cooling (Moran, 2012; Renaudeau et al., 2012; Fournel et al., 2017). The current study was conducted under conditions likely to induce heat stress - in autumn when the THI ranged from 71.9 to 85.6 units, which is considered hot to very hot for the cows (Zimbleman et al., 2009). These results consolidate those in our previous publication (Bang et al., 2021c), which used the same housing management data as the current study and indicated that increased altitude, floor area per cow, roof heights, and per cent of shed sides open increased ventilation and decreased THI inside the cowshed. Many studies have found that decreased THI was associated with decreased heat stress, which then increased feed intake, milk production, and cow fertility (Preez et al., 1990; Ravagnolo et al., 2000; Bouraoui et al., 2002; Könyves et al., 2017). Thus, our results suggest that building farms in high altitude regions, increasing floor area per cow, and increasing eave and ridge roof heights could be effective strategies to increase milk production of Vietnamese SDF cows.

Mats over concrete flooring are supplied for cows in Vietnam SDFs to make them more comfortable when resting. However, in the current study, it has been found that increasing mat area per cow could be more problematic than beneficial as it was associated with a decrease of 0.41 kg/cow/d in MILK and an increase of 0.5 times in tAI. With respect to this issue, Ashbaugh (2010) reported that many Vietnamese SDF farmers did not provide bedding on top of the concrete floors for cows because of hygiene issues, which then caused mastitis. This might indicate that the current type of mats (mainly polyethylene foam mats) used in SDFs might not be suitable. However, the negative association between mat area and MILK might indicate that mat area is an indicator of other variables that were not included in the model.

### Nutritional variables

Increased DMIbw and dietary DM concentration were associated with increased MILK, ECM, ECMbw, and mRE and with decreased tAI, which are all desirable. The cows were in heat-stressed conditions, and the feed intake of heat-stressed cows is commonly depressed as an adaptive response of cows to reduce metabolic heat production (Gaughan and Mader, 2009; Renaudeau et al., 2012). Consequently, a decrease in feed intake was expected, and the decrease in feed intake explains the decrease in milk production associated with cows in heat-stressed conditions (Knapp and Grummer, 1991; West, 2003; West et al., 2003). Although the mechanisms that mediate the effect of heat stress on the reduced milk yield can be multifactorial, at least half of the reduction in milk yield can be explained by the decrease in feed intake caused by heat stress (Tao et al., 2020). The feed intake can be improved by using more concentrates or supplying cows with higher-quality grasses or roughage (less fibre) instead of the current roughage such as Napier grass or rice straw that farmers commonly used for their cows (Chu et al., 2005; Lam et al., 2010; Phong and Thu, 2016). Reducing dietary fibre results in decreasing bulk density of the diet, thus encouraging intake (West, 2003; Renaudeau et al., 2012). Cummins (1992) reported that during heat stress, dry matter intake and milk yield of cows given diets containing 14% to 17% ADF (DM basis) were higher than those of cows offered diets containing 21% ADF.

Increased dietary fat concentrations were associated with increased MILK, ECM, ECMbw, and BCS and associated with decreased PS, which are all desirable. These results are in line with those of other researchers who reported that increasing dietary fat was an effective strategy to eliminate the effects of heat stress on the dairy cow during summer. Lactating cows commonly experience negative energy balance during heat stress (West, 2003; Shwartz et al., 2009). This is because heat-stressed dairy cows are often unable to consume enough feed to meet their energy demands during lactation (Gaughan and Mader, 2009; Renaudeau et al., 2012). As a result, they typically mobilize their body reserves to maintain their milk production until the intake of feed can match or exceed their nutritional requirements (West, 2003). Therefore, a strategy to feed cows during heat stress is to increase dietary nutrient density, especially energy density by supplementing their diets with fat or increasing concentrate to compensate for the decline in feed intake.

In contrast to fat, increased dietary NFC, supplied mainly by the concentrate component of the diet, was negatively associated with mFA, mPR, ECM, and ECMbw and positively associated with PS and tAI. These results were hard to explain because NFC is also a main source of NEL and so is contrary to our hypothesis. However, consistent with our results are those of Drackley et al. (2003), who conducted a study to compare productivity, and heat stress level of HOL cows offered either a control diet (fat: 2.63% DM, NFC: 40.76% DM), high-fat diet (fat: 6.04% DM, NFC: 38.10% DM), or high NFC diet (fat: 2.70% DM, NFC: 46.26% DM) during summer in the midwest of the USA. This study showed that mFA, fat corrected milk yield (3.5% fat), and the efficiency of milk production of the cows offered the high-fat diet were higher than those of cows offered the high NFC diet (Drackley et al., 2003). Respiration rate and rectal temperature were also lower in cows provided with high-fat diets than in cows provided with high NFC diets (Drackley et al., 2003).

Higher dietary lignin was associated with higher PS (more heat-stressed cows), which was to be expected. Diets rich in lignin are also likely to be rich in ADF (Adesogan et al., 2019). In the current study, dietary lignin was also and positively correlated with dietary ADF (r = 0.68, P < 0.05) and negatively associated with dietary NEL (r = −0.56, P < 0.05). Generally, the heat increment from the metabolic utilization of dietary fibre is considered higher than that from the metabolic utilization of starch or fat because the fermentation of fibre generates more heat and is less efficient (Renaudeau et al., 2012). Thus, under heat stress conditions, high fibre diets make cows more heat-stressed. It has also been suggested that feeding cows a diet containing high-quality NDF (low lignin concentration and high digestibility) instead of a diet with low-quality NDF can reduce the negative effects of heat stress on their productivity, body temperature, and feeding behaviour (Arieli et al., 2004; Soriani et al., 2013). Interventions that specifically reduce the lignin but not necessarily the NDF concentration in SDF cows’ diets should be targeted for future research.

Although dietary Na concentration was not associated with either ECM or ECMbw, each per cent (DM basis) increase in dietary Na concentration was associated with a decrease of up to 6 kg of MILK per cow per day. This is inconsistent with the results of Schneider et al. (1986), who reported that increased dietary Na was associated with increased feed intake and milk yield of cows in hot weather. In Vietnamese SDFs, there could be two possible explanations for the negative associations between dietary Na and milk yields. Firstly, cows in Vietnam may not be supplied with enough drinking water during hot weather. Water is crucial for milk excretion, and the need for water increases when dietary Na is increased, especially during hot weather (West, 2003; Meyer et al., 2004; Appuhamy et al., 2016). Two studies on SDFs in Vietnam showed that 51% of SDFs provided less than 30 l of water for a cow per day, while only around 29 to 35% provided fresh water *ad libitum* for the cows (Suzuki et al., 2006; Lam et al., 2010). Our results in previous study (Bang et al., 2021c) also showed quite similar results. A second explanation for the negative associations between dietary Na and milk yields is that during the current study, it was observed that, in the hope of increasing feed intake, Vietnamese SDFs farmers commonly supplemented Na in the form of NaCl rather than NaHCO_3_, whereas a study by Coppock et al. (1982) showed that NaHCO_3_ is likely to be more effective than NaCl in increasing cows’ feed intake and milk yield during heat stress. Similarly, Gaughan and Mader (2009) found that during hot conditions, adding NaCl to the diet can decrease the dry matter intake of cattle. Therefore, the dietary Na finding suggests the importance of research that tests the effect of *ad libitum* versus restricted provision of drinking water on cows in Vietnamese SDFs.

Each per cent increase in dietary P concentration was associated with an increase of up to 4.3 units in PS. To our knowledge, evidence of an association between dietary P and heat stress has not previously been reported. This association might reflect the effects of dietary CP on PS. Crude protein was not included in the PS model due to its variance inflation factor ≥ 10, but the correlation coefficient between P and CP was high. The CP concentration of the diet (16.5% DM) was also in excess relative to energy concentration (1.4 MCal/kg DM) when compared with the concentrations suggested by NRC (2001). When cows are offered diets with excess protein, the excess protein leads to an increase in metabolic heat generated during the excretion of excess nitrogen as urea (Huber et al., 1994; Dunshea et al., 2013), which then might cause an increase in PS.

### Animal variables

A higher genetic proportion of BSW in the herds was associated with increases in MILK, ECM, ECMbw, and BCS, which are all desirable. These results were consistent with the results of other researchers. A study by El-Tarabany et al. (2017) reported that pure BSW and F1HOL_BSW cows are more adaptable to the subtropical conditions in Egypt than pure HOL cows, as shown by a slower rate of reduction in milk yield when THI changed from a low to a high level. That study also showed that F1HOL_BSW had a lower incidence of clinical mastitis, metritis, and feet problems than pure HOL and that pure BSW cows had a lower incidence of retained placenta and metritis than pure HOL (El-Tarabany et al., 2017).

A higher genetic proportion of JER was not associated with milk productivity traits but was associated with decreases in HG or BW and BCS. This was expected because mature BW of JER (408 to 454 kg) is often smaller than mature BW of HOL (590–680 kg) and BSW (509–537 kg) (Capper and Cady, 2012; Piccand et al., 2013).

A higher genetic proportion of ZEB was associated with decreases in mPR, ECMbw, HG, and BW. The negative association between the genetic proportion of ZEB with ECMbw was consistent with the findings of a study by Branton et al. (1961), who reported that in a hot and humid climate, all crossbreeds of HOL with Red Sindhi (a ZEB breed) and crossbreeds of BSW with Red Sindhi yielded less milk and fat compared with their pure HOL or pure BSW mates during both winter and summer. Similarly, the negative associations between the genetic proportion of ZEB with HG and BW are understandable because the mature BW of Vietnamese ZEB cattle were very small (249–281 kg) (Duy et al., 2013). A study by Branton et al. (1961) also showed that the mean BW of F1HOL_RSI (1/2 HOL + 1/2 Red Sindhi) and B1HOL_RSI (3/4 HOL + 1/4 Red Sindhi) crossbred cows were smaller than the BW of pure HOL at various ages from birth to 90 days postpartum.

Thus, the associations between the genetic proportion of breeds and the productivity and welfare indicators in the current study suggest that increasing the genetic proportion of BSW rather than increasing the genetic proportion of ZEB could be a more appropriate breeding strategy for Vietnamese SDFs. Unfortunately, HOL were excluded from the models due to VIF > 10; thus, it was impossible to infer the effects of HOL genetic proportions directly.

The strong positive association between BCS and PS is consistent with a study by Cincović et al. (2011), who reported that high BCS cows (BCS > 4, 5-point scale) had less ability to acclimatize to heat stress than normal and thin cows, indicated by lower milk yield and quality and higher rectal temperature and respiration rate compared to other groups. Brown-Brandl et al. (2006) also reported that finished feedlot Angus and Charolais cattle with BCS ≥ 8 (9-point scale) had an average 6.8% higher respiration rate than the lean animals with BCS < 8.

### Some limitations

A principal aim of the current study was to identify potential causes of the low productivity and welfare of Vietnamese SDF cows. However, it should be noted that similar to many epidemiological, public health, or social studies, it is by nature an observational study conducted in an inherently noisy environment rather than a randomized experimental study where the confounders are controlled (Glass et al., 2013). Thus, the associations found in our multivariate regression models may not be causal. An observed association can be due to the effects of one or more of the following: chance (random error), bias (systematic error), confounding, reverse causality, or true causality (Lucas and Mcmichael, 2005; Barratt et al., 2009). To minimize these negativities, we applied two strategies. Firstly, we collected as many biologically and theoretically sound independent variables as we could to be included in each model (Glass et al., 2013). Secondly, to judge whether an observed statistical association represents a cause-effect relationship between explanatory and response variables (Ward, 2009; Geneletti et al., 2011), we applied as many as possible of Hill’s (1965) criteria that are commonly applied to epidemiological studies. These criteria include the strength of association, consistency, specificity, temporality, biologic gradient, plausibility, analogy, coherence, and experiment (Hill, 1965). According to this method, briefly, explanations from the literature were searched and considered for each observed association to infer the likelihood of that association being a true cause-effect relationship. Then, intervention strategies were suggested based only on the most likely cause-and-effect relationships between explanatory variable and outcome variables. In the current study, we did not consider the associations between dietary NFC with milk production, between dietary P with PS, and between tAI and DIM as causal associations. Thus, further studies are needed to find clear explanations for these associations.

It also should be noted that changes in variables that could have casual effects need to be large enough to be of practical significance. For example, regarding the effect of altitude, each 100 m increase in altitude was associated with a 0.2-kg increase in ECM. So, when considering regions in which to build cowsheds, one region must be some hundred metres higher than the others to have significant benefit. Similarly, each metre increase in cowshed ridge height was associated with a 0.67-kg increase in ECM, so an increase of 2 to 3 m in ridge height could significantly improve ECM. Further, some effects appeared to be significant but had high standard errors, for example, the effects of eave height on mDM, mat area per cow on BW, or floor area per cow on PS. Thus, the impact of changing those variables in the targeted production and welfare parameters remains uncertain. Therefore, the interventions’ cost-effective and practical perspectives need to be considered, but these perspectives were not included in the current study.

In addition, the current study built the multivariate models only for some selected production and welfare indicators. Many other important production and welfare indicators such as reproduction parameters, mobility, and cow cleanness were not studied. Also, due to the limited number of observations, only 32 SDFs, this study did not consider interactions between the predictor variables.

## Conclusion

For Vietnamese SDFs, the following research priorities were identified. Improving shed design to cool the cows ranked as the foremost opportunity to simultaneously improve milk production and cow welfare. Shed improvement research should target reductions in ambient temperature and increased airflow. If possible, build farms in highland regions, but even in highland regions, responses to increases in eave and ridge roof heights, percentage of shed side open, and floor area per cow require testing. Next are dietary interventions. Research needs to target increased dietary dry matter intake and the effects of increased dry matter and fat concentration in the diet on milk production. Research is also needed to test whether a decrease in dietary lignin and phosphorus concentrations can reduce panting score without reducing cows’ milk yield and milk fat concentration. Finally, genetic research should test whether increasing the genetic proportion of BSW but not ZEB in individual cows can improve milk production.

## Data Availability

The data presented in this study are available on request from the corresponding author.

## Appendix

**Table 7:** Pearson correlations between variables included in the models^A,^^B^

Included variables	Altitude	FloorCow	MatCow	RidgeHei	EaveHei	SideOpen	DMIbw	DM	Fat	NFC	Lignin	Na	P	ZEB	BRW	JER
Altitude	1															
FloorCow	0.51**	1														
MatCow	0.04	0.37*	1													
RidgeHei	−0.16	0.1	−0.09	1												
EaveHei	−0.28	0.13	0.1	0.59***	1											
SideOpen	0.34	0.28	−0.11	0.07	0.06	1										
DMIbw	0.15	−0.03	−0.02	0.12	0.04	0.06	1									
DM	−0.06	0.07	−0.1	0.34	0.31	−0.08	−0.04	1								
Fat	−0.15	−0.33	0.04	0.13	0.12	−0.26	−0.09	−0.02	1							
NFC	0.24	−0.06	−0.27	0.33	0.11	0.32	0.18	0−.36*	−0.03	1						
Lignin	0.16	0.62***	0.37*	0.01	0.28	−0.02	0.05	0.14	−0.36*	−0.38*	1					
Na	0.21	0.06	−0.21	−0.08	0.01	0.06	−0.24	0.08	−0.13	0.08	−0.02	1				
P	0.27	0.24	−0.12	−0.01	−0.09	−0.18	−0.03	0.55**	−0.02	−0.08	0.05	−0.12	1			
ZEB	−0.54**	−0.52**	−0.1	−0.18	−0.14	−0.33	−0.04	−0.21	0.18	−0.31	−0.26	−0.19	−0.05	1		
BRW	0.18	0.16	0.09	0.04	0.16	−0.13	−0.14	0.13	0.18	0.05	0.14	0.24	0.29	0.08	1	
JER	−0.33	−0.19	−0.2	0.03	0.19	−0.04	0.08	0.05	0.09	0.06	0	0	0.04	0.04	0.29	1
DIM	−0.13	0.03	0.12	0.02	0.03	−0.08	−0.42*	0.03	0.02	−0.02	0.03	0.03	−0.01	−0.16	−0.15	−0.13
Lactations	0.5**	0.11	−0.17	−0.01	0.06	0.25	0.12	−0.01	−0.17	0.18	0.05	0.14	0.17	−0.08	0.15	−0.26
BW	0.34	0.49**	0.36*	0.17	0.32	0.28	−0.2	0.13	−0.13	0.06	0.26	0.15	0.04	−0.42*	−0.01	−0.42*
BCS	−0.32	−0.1	0.2	0.17	0.35*	−0.07	−0.23	0.1	0.27	0.02	−0.08	−0.04	−0.16	0.2	0.18	−0.21
PS	−0.59***	−0.33	0.02	−0.19	−0.12	−0.5**	−0.09	0.19	−0.11	−0.25	0.06	−0.24	0.18	0.49**	−0.07	0.17
HG	0.34	0.49**	0.37*	0.17	0.31	0.28	−0.22	0.11	−0.11	0.03	0.25	0.16	0.04	−0.4*	0	−0.44*
MILK	0.51**	0.58***	0.19	0.29	0.33	0.2	0.31	0.44*	0.04	0.22	0.3	−0.01	0.44*	−0.38*	0.41*	−0.1
ECM	0.46**	0.46**	0.14	0.32	0.38*	0.23	0.24	0.43*	0.05	0.21	0.27	0.06	0.4*	−0.34	0.47**	−0.03
ECMbw	0.27	0.21	−0.02	0.29	0.36*	0.1	0.45**	0.41*	0.18	0.2	0.19	−0.08	0.41*	−0.16	0.54**	0.25
mFA	−0.6***	−0.38*	−0.05	−0.07	−0.05	−0.09	−0.44*	−0.21	−0.02	−0.39*	−0.08	0.13	−0.27	0.41*	−0.17	0.1
mPR	−0.35	0.06	0.28	0.03	0.3	0.08	−0.36*	−0.05	0.04	−0.26	0.23	0.03	−0.28	−0.23	−0.06	0.09
mDM	−0.58***	−0.52**	−0.06	−0.13	−0.03	−0.04	−0.17	−0.36*	−0.1	−0.32	−0.09	0.07	−0.43*	0.49**	−0.05	0.2
mRE	0.6***	0.17	0.01	0.04	−0.07	0.38*	0.36*	−0.19	−0.03	0.34	−0.07	0.19	−0.14	−0.43*	−0.04	−0.1
tAI	−0.4*	−0.16	0.18	−0.11	−0.1	−0.29	−0.27	0	−0.24	0.04	−0.11	−0.12	−0.11	0.21	−0.09	0.09
Included variables	DIM	Lactations	BW	BCS	PS	HG	MILK	ECM	ECMbw	mFA	mPR	mDM	mRE	tAI		
DIM	1															
Lactations	−0.26	1														
BW	0.11	0.37*	1													
BCS	−0.02	−0.07	0.42*	1												
PS	0.06	−0.25	−0.23	0.24	1											
HG	0.12	0.37*	1***	0.43*	−0.23	1										
MILK	−0.31	0.42*	0.52**	0.1	−0.27	0.52**	1									
ECM	−0.29	0.46**	0.57***	0.21	−0.21	0.57***	0.95***	1								
ECMbw	−0.42*	0.37*	0.16	0.09	−0.08	0.15	0.85***	0.88***	1							
mFA	0.35*	−0.25	0	0.28	0.52**	0.02	−0.58***	−0.39*	−0.45*	1						
mPR	0.32	−0.31	0.3	0.35	0.13	0.31	−0.12	−0.05	−0.15	0.45*	1					
mDM	0.04	−0.19	−0.17	0.22	0.47**	−0.16	−0.58***	−0.4*	−0.35	0.81***	0.39*	1				
mRE	−0.03	0.38*	0.21	−0.3	−0.46**	0.22	0.29	0.28	0.2	−0.27	0.02	−0.17	1			
tAI	0.5**	−0.46**	−0.35*	−0.15	0.26	−0.35*	−0.52**	−0.58***	−0.53**	0.18	0	0.15	−0.31			

^A^Significant levels were adjusted by the Bonferroni method: **P* <0.05, ***P* <0.01, and ****P* <0.001

^B^Abbreviations: Altitude, (m); FloorCow, floor area per cow (m^2^/cow); MatCow, mat area per cow (m^2^/cow); RidgeHei, ridge roof height (m); EaveHei, eave roof height (m); SideOpen, per cent of shed sides open (%); DMIbw, dry matter intake per body weight (% BW); DM, dietary dry matter concentration (%); Fat, dietary fat (% DM); NFC, dietary non-fibre carbohydrate (% DM); Lignin, dietary lignin (% DM); Na, dietary sodium (% DM); P, dietary phosphorus (% DM); ZEB, average zebu ancestry proportion (%); BRW, average Brown Swiss ancestry proportion (%); JER, average Jersey ancestry proportion (%); DIM, days in milk; Lactations, number of lactations; HG, heart girth (cm); BW, body weight (kg); BCS, body condition score (1 to 5); MILK, milk yield (kg/cow/d); ECM, energy-corrected milk (kg/cow/d); ECMbw, ECM per 100kg of body weight (kg/100 kg BW/d); mFA, milk fat concentration (%); mPR, milk protein concentration (%); mDM, milk dry matter concentration (%); mRE, milk electrical resistance (units); PS, panting score (0 to 4.5); tAI, inseminations per conception (times)
